# Differential Transcriptomic Response of Rainbow Trout to Infection with Two Strains of IPNV

**DOI:** 10.3390/v14010021

**Published:** 2021-12-23

**Authors:** David Tapia, Juan Kuznar, Rodolfo Farlora, José M. Yáñez

**Affiliations:** 1 Facultad de Ciencias Veterinarias y Pecuarias, Universidad de Chile, Santiago 8820000, Chile; davidtapiae@gmail.com; 2 Doctorado en Acuicultura, Pontificia Universidad Católica de Valparaíso, Universidad Católica del Norte, Universidad de Chile, Valparaiso 2340000, Chile; 3 Laboratorio de Virología, Facultad de Ciencias, Instituto de Química y Bioquímica, Universidad de Valparaíso, Valparaiso 2340000, Chile; juan.kuznar@uv.cl; 4 Laboratorio de Biotecnología Acuática y Genómica Reproductiva, Instituto de Biología, Facultad de Ciencias, Universidad de Valparaíso, Valparaiso 2340000, Chile; rodolfo.farlora@uv.cl; 5 Centro de Investigación y Gestión de Recursos Naturales (CIGREN), Universidad de Valparaíso, Valparaiso 2340000, Chile; 6 Center for Research and Innovation in Aquaculture (CRIA), Universidad de Chile, Santiago 8820000, Chile

**Keywords:** IPNV, rainbow trout, transcriptome, RNA-Seq

## Abstract

The IPN virus (IPNV) causes a highly contagious disease that affects farmed salmonids. IPNV isolates have been phylogenetically classified into seven genogroups, of which two are present in Chile, genogroups 1 and 5. This study aimed to compare the transcriptomic response of rainbow trout fry challenged with two Chilean isolates of IPNV, RTTX (genogroup 1), and ALKA (genogroup 5). Tissue samples from challenged individuals and controls were taken at 1, 7, and 20 days post-challenge and analyzed by RNA-Seq. The results revealed that infection with RTTX elicited a greater modulation of the trout transcriptome compared to ALKA infection, generating a greater number of highly differentially expressed genes in relation to the control fish. Gene Ontology enrichment indicated that functions related to the inflammatory and immune responses were modulated in fish challenged with both isolates throughout the trial, but with different regulation patterns. On day 1 post challenge, these functions were activated in those challenged with ALKA, but suppressed in RTTX-challenged fish. These results suggest that rainbow trout exhibit a differential transcriptomic response to infection with the two genetically distinct IPNV isolates, especially at early times post-infection.

## 1. Introduction

Rainbow trout (*Oncorhynchus mykiss*) is one of the most widely farmed finfish in the world. The species is cultivated in more than seventy countries and currently the main producers are Turkey, Iran, Norway, Chile, and Peru [1]. Infectious pancreatic necrosis (IPN), a highly contagious viral disease that affects salmonid fish in farming systems including rainbow trout [2,3], has been found in all major producer countries [4,5,6,7,8]. IPN outbreaks occur mainly during the freshwater stage in first-feeding fry, and to a lesser extent in post-smolts after transfer to the sea [9]. Cumulative mortalities during outbreaks can vary greatly, with reports of up to 100% in young fry, and between 10 to 20% in post-smolts [9]. Fish that survive can become asymptomatic carriers and transmit disease both horizontally to other susceptible fish or vertically to their offspring [10]. 

The cause of the disease is the IPN virus (IPNV), the type species of the genus *Aquabirnavirus*, belonging to the *Birnaviridae* family, characterized by having a bi-segmented double-stranded ribonucleic acid (RNA) genome [11]. IPNV and other aquabirnaviruses have traditionally been classified into two serogroups, A and B, consisting of nine and one serotypes, respectively [12]. Currently, IPNV isolates are classified primarily based on the genetic sequence of the virus capsid protein, VP2. Phylogenetic analyses have shown that aquabirnaviruses can be grouped into seven genogroups [12,13]. These genogroups correlate well with the traditional serological classification and geographical origin of the archetype IPNV strains. For example, reference strains from the United States classified serologically as serotype 1 such as West Buxton (WB) and VR-299 form part of genogroup 1, while the archetypal European strains, Sp and N1, traditionally classified as serotype 2, belong to genogroup 5. Isolates belonging to these two genogroups are the most common and prevalent in salmonid producing countries around the world [14].

As with any viral disease, several host- and virus-related factors are associated with the onset, development, and severity of an IPN outbreak [14]. It is well known that, in addition to host age and species, genetic differences in resistance/susceptibility to the virus and virulence determinants in IPNV isolates are key factors affecting disease outcome [2,14,15]. For instance, a major quantitative trait locus (QTL) for resistance against IPN has been identified in Atlantic salmon [16,17], and studies show that there is also significant genetic variation for IPN resistance in rainbow trout [18,19]. Whereas the viral gene that codes for the VP2 protein is attributed a very important role in the virulence of IPNV [20,21,22,23]. In addition, recent evidence in rainbow trout suggests that virulence determinants found in IPNV are strain dependent [5,24,25].

To better understand some of the predisposing factors of IPN outbreaks, several studies have analyzed the transcriptomic response of IPNV-challenged fish using high-throughput technologies such as cDNA expression microarrays. For example, Skjesol et al. [26] analyzed the response of Atlantic salmon fry challenged with high and low virulence IPNV isolates. Microarray analysis at day 13 post-challenge revealed a greater number of differentially expressed genes (DEGs) in the head-kidney of salmon infected with the most virulent isolate, with several antiviral response genes (e.g., antigen presentation and interferon induced genes) only upregulated in these fish. This was in turn correlated with higher viral load and greater mortality. On the other hand, studies comparing the transcriptomic response of genetically resistant and susceptible Atlantic salmon to IPNV have detected marked differences in the gene expression profiles between fish of both phenotypes when challenged with the virus [27,28]. In general, it seems that susceptible fish mount an exacerbated but short early immune response, with a marked overexpression of inflammatory genes, while resistant fish show a more moderate and constant immune response that they manage to sustain over time. However, there have been no studies that analyze the transcriptomic response of rainbow trout to infection with IPNV, let alone to infection with different strains of the virus. In addition, only a few studies have analyzed the whole transcriptome of fish challenged with IPNV using RNA-Seq [29,30].

In a previous study, we showed that viral load and mortality varied in rainbow trout fry challenged with Chilean IPNV isolates from genogroups 1 and 5, more commonly known in the country as WB and Sp strains, respectively [25]. Continuing with that research, here, we compared the transcriptomic response of the challenged rainbow trout individuals using RNA-Seq to determine whether there was a differential gene expression in response to infection with isolates from the two IPNV genogroups. Fish challenged with a genogroup 1 isolate and a genogroup 5 isolate were sampled on days 1, 7, and 20 post-challenge and contrasted with time-paired mock challenge control fish. RNA-Seq and differential gene expression analysis (DGE) was performed to investigate the differences in global gene expression patterns between fish challenged with both IPNV isolates. Furthermore, Gene Ontology (GO) analysis was carried out to evaluate the functions associated with differentially expressed genes in order to better understand host response to infection to different strains of IPNV.

## 2. Materials and Methods

### 2.1. Virus, Fish and Experimental Challenge

Two IPNV isolates were used for the experimental challenge in this study: RTTX from genogroup 1 and ALKA from genogroup 5. The isolates were obtained during IPN outbreaks in Chile and belong to the ceparium of the Virology Laboratory at the Universidad de Valparaíso. Both isolates had a single passage in cell culture and had previously been molecularly characterized by sequencing of their entire genome, as described by Jorquera et al. (2016) [31]. Isolates were propagated in monolayers of Chinook Salmon embryo cell line CHSE-214, grown in Leibovitz (L-15) culture medium, supplemented with 10% fetal bovine serum (FBS, HyClone) and 50 μg·mL^−1^ gentamicin, following the methodology described by Espinoza and Kuznar [32]. The viral inocula were titrated by a standard end-point dilution assay in CHSE-214 cells maintained in 96-well plates and TCID_50_ was calculated according to the method of Reed and Muench [33].

Rainbow trout fry (~0.9 g) were provided by Rio Blanco fish farm (Los Andes, Chile) and transferred to the aquaculture facilities of the “Laboratorio Cerrillos”, Veterquimica S.A., where they were challenged. Prior to the challenge, the fish were checked for the presence of IPNV, *Renibacterium salmoninarum, Piscirickettsia salmonis, Flavobacterium psychrophilum,* and *F. columnare* by qPCR at the Veterquimica diagnostic laboratory. Fish were held in fresh water at 10–15 °C during 22 days for acclimatization and fed daily on commercial feed until the day preceding virus exposure.

After acclimatization, the fish were distributed in 20 L tanks with around 180 individuals each. Once in their respective tanks, the temperature was adjusted to 10 °C and the individuals were challenged by immersion with stopped water flow using the IPNV isolates at a predicted dose of 1 × 10^5^ TCID_50_/_mL_ for 3 h. In the control group, the challenge was simulated by adding virus-free culture medium L-15 to the tank. Subsequently, the fish were kept at 10 °C and mortality was recorded daily until the trial ended 30 days post-challenge. Three live fish were sampled from each tank (challenged and controls) on days 1, 7, and 20 post-challenge. Fish were euthanized using benzocaine overdose (BZ-20; Veterquimica), weighed and tissue samples were taken (headless fish without tail or fins) and kept in RNAlater^®^ solution (Ambion, Austin, Texas, United States) at −80 °C until processing. The presence of IPNV and viral load were analyzed in the samples using an RT-qPCR assay as described by Tapia et al. [25].

More detailed description of the virus preparation and the experimental challenge are given in Tapia et al. [25], along with the mortality reports from the trial. Briefly, 1440 individuals were transferred to research station, acclimatized for three weeks, and then distributed into eight 0.02 m^3^ tanks, with approximately 180 fish per tank. Two tank replicates per treatment were used. The challenged was carried out by immersion, using IPNV isolates from genogroups 1 and 5 at 1 × 10^5^ TCID_50_/_mL_. Animals were kept at 10 °C and mortality was collected daily until 30 days post-infection. All the procedures for the challenge and sampling were approved by the Animal Bioethics Committee, Faculty of Veterinary and Livestock Sciences, Universidad de Chile (Certificate No. 17086-VET-UCH).

### 2.2. RNA-Seq and Bioinformatic Analysis

To compare the transcriptomic response of rainbow trout challenged with the two IPNV isolates, tissue samples of the fish at days 1, 7, and 20 post-challenge (dpc) were analyzed using RNA-Seq. At each time point, ALKA and RTTX challenged fish were contrasted to mock challenge control fish used as a common reference (three individuals from each group). The analyzed tissues corresponded to the complete viscera (including head kidney, liver, spleen, and intestines) together with fish muscle, and were homogenized in 1 mL of TRIzol reagent (Invitrogen, Waltham, Massachusetts, United States) using homogenization beads. Total RNA was extracted following the standard RNA isolation procedure with TRIzol. From the extracted RNA, RT-qPCR tests were carried out targeting both the VP1 protein gene to detect the presence of the virus and the alpha elongation factor (ELF1α) gene of fish, which is used to evaluate the condition of the tissue samples, and as an endogenous control for the normalized quantification of VP1, as described by Tapia et al. [25]. The RNA concentration was measured in a Quantus fluorimeter (Promega, Madison, Wisconsin, United States) and the integrity was analyzed using 1% agarose gel electrophoresis, following the recommendations of Aranda et al. [34]. To determine the quality of the RNA using this method, the presence of two clear and sharp bands of rRNA 28S (~4.8 kb) and 18S (~2.0 kb) was considered. 

The RNA was precipitated with ethanol and samples were sent with ice gel packs (ice-packs) to Macrogen Inc. (Seoul, South Korea), where the RNA-Seq libraries were built and sequenced. Prior to sequencing, samples were subjected to quality control where the integrity and quantity of the RNA were verified on a 2100 Bioanalyzer platform (Agilent Technologies, Santa Clara, California, United States). All samples presented an RNA integrity number (RIN) greater than 8. A total of 27 cDNA libraries were built, considering three conditions (RTTX challenged, ALKA challenged, and control), three biological replicates per condition (one individual each), and three times (1, 7, and 20 dpc) using the TruSeq Stranded mRNA LT Sample Prep Kit (Illumina, San Diego, California, United States). The sequencing of the libraries was performed on an Illumina Novaseq platform (Illumina) with a paired-end sequencing strategy and a read length of 100 bp.

Sequence analysis was performed using the CLC Genomics Workbench Version 12.0 software (CLC bio, Aarhus, Denmark). First, the raw reads were pre-processed by trimming the low quality sequences (limit = 0.05) and the adapter sequences, and by removing the sequences of length < 50 nucleotides. The filtered reads were then mapped to the rainbow trout reference genome (Omyk_1.0, version 100) available from the NCBI database (https://www.ncbi.nlm.nih.gov accessed on 15 December 2018). The parameters used were mismatch penalty (mismatch) = 2, gap penalty (insertion/deletion) = 3, length fraction = 0.8, and similarity fraction = 0.8. The gene expression values were calculated using the transcripts per million parameter (TPM) for the normalization of the count of reads mapped to the genome. A principal component analysis (PCA) was performed to evaluate the similarity between the groups of samples for each condition and time. The differential expression tool for RNA-Seq included in the CLC Genomics Workbench software was used to compare the differential expression of genes (DGE) between the different conditions. Two comparisons were made for each time analyzed: RTTX challenged vs. control and ALKA challenged vs. control. Genes with a *p*-value corrected for multiple comparisons by the false discovery rate (FDR) ≤ 0.01 and an absolute Log2 Fold Change (LogFC) ≥ 2 in relation to the control group were considered differentially expressed genes (DEG). Additionally, differentially expressed genes with absolute LogFC ≥ 4 were visualized using volcano plots.

Venn diagrams were constructed from the DEG lists obtained to identify genes that were up- or downregulated in fish challenged with RTTX as well as ALKA challenged fish at each time point. The diagrams were built on the online platform: http://www.venndiagrams.net (accessed on 6 June 2020) and the lists of genes shared between the fish infected with both isolates were obtained using the BioInfoRx tool “Data Overlapping and Area-Proportional Venn Diagram” (http://apps.bioinforx.com/bxaf6/tools/ accessed on 6 June 2020). Human homologues of the differentially expressed genes were used for the functional enrichment analysis since the functional information of human genes is more extensive and robust than that available for rainbow trout. Homologous genes were identified with the NCBI local sequence alignment (BLAST) search tool (https://blast.ncbi.nlm.nih.gov/Blast.cgi, accessed on 11 June 2020). To do this, the identifiers of the differentially expressed genes: “Gene ID” were converted to “RefSeq Protein Accession” in the biological DataBase network (bioDBnet) platform (Mudunuri et al., 2009) [35] with the tool “Conversions from databases to databases” (https://biodbnet-abcc.ncifcrf.gov/db/db2db.php, accessed on 11 June 2020). Subsequently, a protein–protein search (BLASTp) was performed against the “Annotated proteins” database of the latest version (annotation 109) of the Homo sapiens genome (taxid: 9606) using an *E*-value of 1e-5 as a threshold. The identifiers of the human proteins were obtained, selecting only those that had the highest alignment score, considering the highest coverage percentage and the lowest *E*-value. A Gene Ontology (GO) functional enrichment analysis was performed using the ShinyGO v0.61 web application [36] (http://bioinformatics.sdstate.edu/go/#tab-4403-10, accessed on 15 July 2020). The enriched GO terms associated with biological processes were detected considering a cut-off *p*-value (FDR) of 0.05, which was calculated using a hypergeometric distribution, followed by a correction using FDR. Finally, bubble graphs with the main GO terms enriched for each time point were built using R version 4.0.2 (R Foundation for Statistical Computing, Vienna, Austria) and the R package ggplot2 [37].

## 3. Results

Mortality results of the challenge with the two IPNV strains were presented in a previous work [25]. Overall, mortality was low in the RTTX-challenged group, but significantly higher than in the ALKA-challenged fish, which showed almost no virus-caused mortality. However, samples taken from live fish showed that trout from both groups exhibited IPNV viral load, which was even higher in ALKA challenged fish at 20 dpc. Table 1 shows the Ct values for the RT-qPCR assay used to estimate IPNV viral load in the samples analyzed in this study. On day 1 post challenge, the presence of IPNV was not detected in any of the fish samples tested. At 7 dpc, viral load was detected only in fish challenged with RTTX, while at 20 dpc, IPNV was detected in trout challenged with both RTTX and ALKA.

After sequencing the cDNA libraries, 1,409,653,870 total reads were obtained out of which 1,409,550,071 high-quality reads (99.99%) were identified, with an average of more than 50 million reads per library. In the PCA, the sum of the PC1 and PC2 components explained 29.3% of the total variability of the dataset (Figure S1). The analysis revealed a pattern of separation between the samples mainly associated with the sampling time and, to a lesser extent, with the condition in the challenge. The control samples presented were more grouped, while those challenged with the isolates were more dispersed, probably reflecting the individual variation of the fish in response to infection with IPNV.

**Figure 1:**
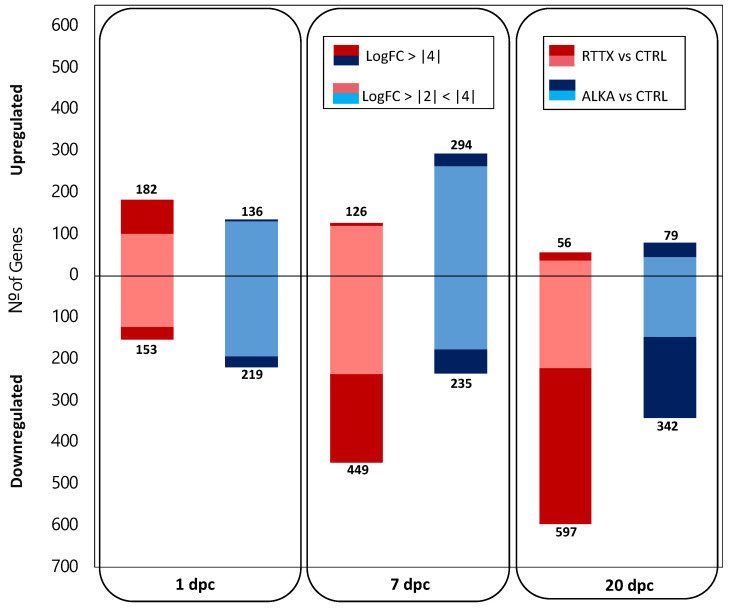
Number of differentially expressed genes (DEGs) in fish challenged with RTTX (pink and red) and ALKA (light blue and blue) when compared to the controls on days 1, 7, and 20 post-challenge. The DEGs were determined considering a Log2 Fold Change (LogFC) ≥ |2| and a *p*-value corrected for the false discovery rate (FDR) ≤ 0.01. Light tones (pink and light blue) show the genes with a LogFC ≥ |2| and <|4|, darker tones (red and blue) indicate the DEGs that presented a LogFC ≥ |4|.

Differential gene expression (DGE) analysis indicated that during the early stages of infection, both IPNV isolates generated a similar number of genes significantly differentially expressed in challenged trout when contrasted to uninfected control fish used as a common reference (FDR ≤ 0.01 and LogFC ≥ |2|) (Figure 1; Supplementary File S1). At 1 dpc, a total of 335 and 355 differentially expressed genes (DEGs) were detected in fish challenged with RTTX and ALKA, respectively; while at 7 dpc, 575 and 529 DEGs were detected in both groups, respectively. On the other hand, in the late stage of infection (20 dpc), trout challenged with RTTX had a considerably higher number of DEGs (653) than those challenged with ALKA (421). 

More interestingly, different patterns in the number of up- and downregulated genes were observed in both groups of challenged fish at the first timepoints analyzed. For example, at 1 dpc, more genes were upregulated (182) than downregulated (153) in the fish challenged with RTTX; meanwhile, ALKA-challenged trout had a greater number of downregulated genes (219) than upregulated ones (136). At 7 dpc, an inverse pattern was observed in the number of DEGs: fish challenged with RTTX had a greater number of downregulated genes (449) than upregulated (126); while ALKA-challenged fish had more upregulated genes (294) than downregulated (235). Finally, at 20 dpc, in both groups of fish, most of the DEGs were found to be downregulated. However, the number of negatively regulated genes in fish challenged with RTTX was much higher (597) than in those challenged with ALKA (342).

These differences between the responses to both isolates were even more marked when analyzing the DEGs that presented a greater magnitude of change since RTTX generated the modulation of a greater number of genes with LogFC ≥ |4| than ALKA in the three times analyzed (Figure 1). Volcano plots clearly showed a higher DEG number with LogFC ≥ |4| in fish challenged with RTTX, especially when observing the overexpressed genes on day 1 post-infection and those downregulated on days 7 and 20 post-infection (Figure S2).

**Figure 2:**
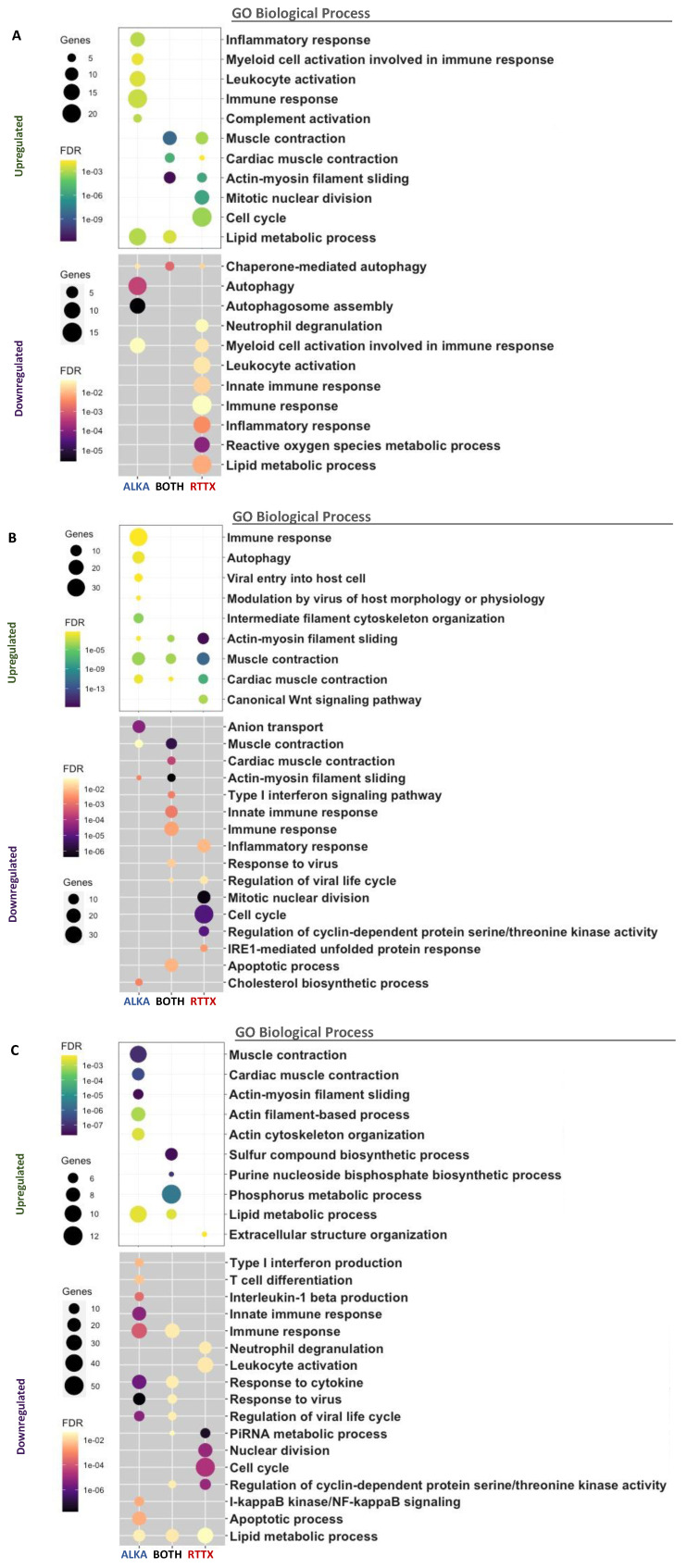
Functional enrichment analysis of differentially expressed genes in fish challenged with RTTX and ALKA at days 1 (**A**), 7 (**B**), and 20 (**C**) post-challenge. Up- and downregulated genes are shown separately for each day. The bubble graphs show the main enriched GO terms associated with biological processes for the genes exclusively regulated in the fish challenged with RTTX or with ALKA, or for the genes shared between the fish challenged with the two isolates (BOTH). The size of the bubble indicates the number of genes mapped to each function and the color the significance of the enrichment (*p*-value corrected by FDR).

To compare the transcriptomic response of trout challenged with ALKA and RTTX, Venn diagrams were constructed with lists of differentially expressed genes (DEGs) (Figure S3) and a Gene Ontology enrichment analysis (GO) was performed using human homologous genes (Figure 2). Venn diagrams of the DEGs showed that several genes were downregulated or upregulated in both RTTX and ALKA challenged fish at the three timepoints analyzed, but especially at 7 and 20 dpc. Similarly, functional enrichment analysis revealed several GO terms repeatedly enriched between upregulated and downregulated genes in response to both IPNV isolates at the different time points analyzed (Figure 2). The main enriched functions at each post-infection time are described below in terms of “biological processes”, both for the genes exclusively regulated in fish challenged with RTTX or with ALKA, and for genes shared between the fish challenged with both isolates according to the Venn diagrams. Additionally, Table S1 details the differentially expressed genes with functions related to the immune response in each group (challenged with RTTX or ALKA) as well as those that were common between both groups (BOTH).

On the first day post-challenge, 50 upregulated genes shared between the fish challenged with both isolates were identified (Figure 2A). The main GO terms enriched among these genes were associated with processes based on actin filaments such as “muscle contraction”, “cardiac muscle contraction”, and “actin-myosin filament slippage”, which were also enriched between the exclusively overexpressed genes in response to RTTX. The term “lipid metabolism processes” was also enriched among the overexpressed genes shared in the fish challenged with both isolates, and also in the genes regulated exclusively by ALKA. The inflammatory response was enriched among genes exclusively overexpressed by ALKA, together with the immune response, which included functions such as “activation of leukocytes”, “activation of complement”, and “activation of myeloid cells involved in the immune response”, driven by the upregulation of genes such as *CD79B, IGLL5, PLA2G3, CD200, CST3, CFP, C4B*, and *CFP* (Table S1). The cell cycle was enriched among genes exclusively overexpressed by RTTX, especially “mitotic nuclear division”. At the same time, the analysis of the downregulated genes allowed us to identify 55 genes shared between the fish challenged with the two isolates (Figure 2A). These genes showed very few enriched terms, the main one being “chaperone-mediated autophagy” which, in addition, was enriched among genes exclusively regulated by RTTX and ALKA. Additionally, “autophagy” and more specifically “autophagosome assembly” were highly enriched among genes exclusively downregulated by ALKA. Among the genes downregulated exclusively by RTTX, an enrichment of the inflammatory response and the immune response was observed. The latter was mainly associated with the “innate immune response”, “activation of myeloid cells involved in the immune response”, and “neutrophil degranulation” with the downregulation of genes such as *SOCS3, NOS2, TLR5, ACOD1, PIK3CG, HEL-S-62p,* and *CEACAM1*.

At seven dpc, an enrichment of the terms related to actin “muscle contraction”, “cardiac muscle contraction”, and “actin-myosin filament slippage” was again observed among the genes upregulated by the two isolates, but especially in genes overexpressed exclusively by RTTX (Figure 2B). In the latter, the “canonical Wnt signaling pathway” was also enriched, related to the regulation of gene transcription. The immune response was enriched among genes upregulated by ALKA, along with the virus-associated terms “viral entry into the host cell” and “virus modulation of host morphology and physiology”, as a result of the modulation of genes such as *MX2, TRIM39, TRIM21, CD209, CLEC4M MRC1*, and *AXL*. At this time, a large number of downregulated genes shared between the fish challenged with the two isolates was identified (191). An enrichment of the terms related to actin and muscle contraction was also observed. The immune response was also enriched in genes downregulated by both isolates, more specifically, the “innate immune response” and the “type 1 interferon signaling pathway”, together with “virus response” and “regulation of the viral life”. These functions were driven by the downregulation of genes such as *STAT1, USP18, RSAD2*, and the interferon inducible genes *HERC5, IFIT5, IFI44L*, and *MX1*. The inflammatory response and the cell cycle were enriched in genes downregulated exclusively by RTTX, together with the function “regulation of the activity of cyclin-dependent protein kinase”, a protein involved in the control of cell division and modulation of the transcription. Among the genes exclusively downregulated by ALKA, the main enriched terms were “anion transport” and “cholesterol biosynthesis processes”.

At 20 dpc, the main enriched terms between the genes overexpressed by both IPNV isolates corresponded to various metabolic processes such as “phosphorus metabolic processes”, “biosynthetic processes of sulfur compounds”, “biosynthetic processes of purine nucleoside”, and “lipid metabolic processes” (Figure 2C). Actin-related terms and muscle contraction continued to be highly enriched among upregulated genes, but this time only in response to ALKA. Very few enriched terms were observed among the genes exclusively overexpressed by RTTX, with “organization of the extracellular structure” standing out only. At this time, a large number of downregulated genes shared between the fish challenged with the two isolates were again identified (191). Among the main terms enriched were “immune response”, “response to cytokines”, “response to viruses”, and “regulation of the viral life cycle” with the downregulation of genes such as *TRIM21, CD209 CLEC4M, ZC3H12A, MRC1, IFI27*, and *IRF5*. These functions were even more enriched among genes negatively regulated exclusively by ALKA, where they also included the “innate immune response”, the “production of interleukin-1 beta”, the “production of type I interferon”, and the “T cell differentiation” with the modulation of genes such as *LGP2, ZC3HAV1, STAT1, RSAD2, NLRC3, TRIM25, HERC5, CCL19, EGR1, IFIT1, IFIT5*, and *MX1*. Enriched in these genes were also “apoptotic processes” and “I-kappaB kinase/NF-kappaB signaling”, a pathway related to the regulation of the inflammatory response. The “lipid metabolic processes” were enriched in genes downregulated by both isolates, but the number was greater in those inhibited in response exclusively to RTTX. The term “cell cycle” continued to be enriched among genes negatively regulated by RTTX, also associated with “regulation of the activity of the protein serine/threonine kinase dependent on cyclin” and “metabolic processes of PiRNA”, related to epigenetic modulation and silencing of genes.

## 4. Discussion

Prior studies have shown differences in the transcriptomic response of salmonids to IPNV when comparing several factors related to mortality caused by the virus. For example, microarray analyses have shown that Atlantic salmon genetically resistant and susceptible to IPN had a clear differential gene expression pattern when challenged with the virus. Similarly, salmon infected with virulent and avirulent isolates of IPNV also showed differential gene expression patterns. Both host genetic resistance/susceptibility to IPN and virulence of IPNV isolates influence viral load and mortality in salmonids. In a previous work, we showed that the mortality and viral load in rainbow trout infected with IPNV varied in relation to the challenging genogroup [25]. That is, trout challenged with genogroup 1 isolates exhibited a slightly higher mortality than those challenged with genogroup 5 viruses, even though the later fish had a higher viral load at different times post-challenge. In the present study, we set out to compare the global gene expression profiles of rainbow trout challenged with IPNV of both genogroups using RNA-Seq. We found that rainbow trout exhibited differential transcriptomic response when challenged with Chilean IPNV isolates RTTX and ALKA, from genogroups 1 and 5, respectively. 

Results showed that infection with both isolates generated changes in the transcriptome of challenged trout in relation to control fish, but to a much greater degree in those challenged with RTTX, which presented higher mortality in the trial. Differential gene expression analysis revealed that the number of differentially regulated genes in relation to the control group was similar between fish challenged with both isolates at 1 and 7 dpc, but higher in fish challenged with RTTX at 20 dpc. However, when considering the differentially expressed genes that presented a higher magnitude of change in relation to the control (LogFC ≥ |4|), RTTX generated the modulation of a greater number of genes at three times post-challenge. The results also showed that the changes generated by both isolates in the trout transcriptome increased as the challenge progressed, especially the downregulation of genes in response to RTTX.

These results differ from previous studies that have analyzed the transcriptomic response of salmon to IPNV, which showed either a similar number of negatively and positively regulated genes, or a prominent number of upregulated genes in fish challenged with the virus [28,29]. For example, genetically susceptible salmon to IPNV presented high mortality levels when challenged with the virus and displayed a high abundance of highly upregulated genes in relation to the control group, especially at times 7 and 20 dpc [28]. However, in salmon challenged with a highly virulent isolate, a greater number of downregulated genes was observed at day 13 post-challenge [26], in accordance with the present results. 

In the present study, differential patterns were observed between the number of upregulated and downregulated genes in rainbow trout challenged with the two IPNV isolates during the early stages of infection, possibly associated with viral load detected in fish. On the first day post-challenge, RTTX produced a higher number of upregulated than downregulated genes, while ALKA generated more downregulated genes. This relationship was reversed at 7 dpc, when viral load and a higher number of negatively regulated genes were detected in fish challenged with RTTX, while fish challenged with ALKA showed more upregulated genes. However, at 20 dpc, both isolates produced a similar pattern of differential expression, with more genes downregulated than upregulated, many of which were shared between fish challenged with both isolates, probably associated with the presence of viral load levels detectable by RT-qPCR in fish of both groups. These results indicate that the differences in the transcriptomic response between the trout challenged with the two isolates were stronger during the early stage of infection, especially one week after challenge, when IPNV viral load could only be detected in fish challenged with the isolate from genogroup 1.

Gene Ontology enrichment analysis indicated that the most represented functions among the modulated genes in response to infection by both IPNV isolates were related to processes based on actin filaments such as muscle contraction (especially cardiac) and cell contraction mediated by actin-myosin slippage. These functions were enriched between up- and downregulated genes in both groups of challenged fish throughout the experiment, but especially one week after the challenge. Previous works have also reported the enrichment of functions related to muscle contraction when analyzing the transcriptomic response of Atlantic salmon challenged with IPNV ([28,29]. However, these functions were only enriched among downregulated genes in virus-challenged salmon, either early (Tarifeño-Saldivia et al., 2018) [29] or late time post-infection (Robledo et al., 2016) [28]. 

Macropinocytosis has been described as the main pathway for IPNV internalization in cells [38,39]. Macropinocytosis is an endocytic mechanism, normally involved in fluid uptake, which is carried out through actin-dependent membrane protrusion and retraction, resulting in large intracellular vacuoles called macropinosomes. The process involves the activation of a complex signaling pathway that transiently modifies the dynamics of the cell’s actin cytoskeleton. Viruses belonging to different families use macropinocytosis as an endocytic pathway for productive infection, and although one of the most common reasons could be the size of the viral particles (as it has been described in predominantly larger viruses), it has been suggested that another reason could be the evasion of the immune response [40]. Studies in cell lines indicated that IPNV induces a rearrangement of the actin cytoskeleton rapidly after inoculation, which is consistent with the macropinocytosis that occurs in infected cells [38,39]. These results are in agreement with the gene expression results obtained in this study, indicating that the modulation of the actin-myosin network in the cells of fish infected with IPNV plays an important role in the productive infection of the virus.

As expected, the inflammatory and immune response were modulated in fish challenged with both isolates of IPNV, but with different regulation patterns throughout the experiment, especially during the early stages of infection. For example, on the first day post-challenge, the inflammatory response and functions related to immune response were enriched among the overexpressed genes in the ALKA-challenged fish. Conversely, these were negatively regulated in those challenged with RTTX, especially the inflammatory response and the innate immune response. These results differ from those of previous studies carried out on families of Atlantic salmon susceptible and resistant to IPNV, where an early acute, highly inflammatory immune response is characteristic of susceptible families with higher levels of mortality [27,28]. In contrast, here, the fish that showed a marked activation in the immune response and the inflammatory response at the beginning of the infection were those that presented the lowest mortality during the challenge (challenged with ALKA), while the fish that presented higher mortality (challenged with RTTX) [25] had a massive downregulation of the immune response the three times analyzed.

The immune response induced in fish challenged with ALKA at first dpc was characterized by the activation of both the leukocytes of myeloid origin and the complement system. The complement system is a vital component of the innate immune response, which acts as a highly complex surveillance system that plays a key role in the defense against pathogens, promoting the inflammatory response and modulating the adaptive immune response. Among the overexpressed genes associated with this function were those that code for the complement component proteins 4B (C4B) and properdin (CFP). Coincidentally, properdin has been reported to be induced, along with other components of complement, in the anterior kidney of rainbow trout alevins immunized with an oral vaccine against IPN [41]. Previous studies in genetically susceptible and IPN-resistant Atlantic salmon have also shown a modulation of the complement system in response to infection with the virus [27,28]. However, both reports address a downregulation of the complement cascade and seem to differ from each other, being detected in resistant fish in early stages of infection (1 and 5 dpc) [27] or in susceptible fish at a late stage (20 dpc) [28].

Meanwhile, at 1 dpc, RTTX generated a negative regulation of genes related to the inflammatory response and the innate immune response, particularly the activation of myeloid leukocytes. Among the genes associated with the immune and inflammatory response, one of the most dysregulated was the Toll-like receptor protein 5 (*tlr5)* gene. Toll-like receptors (TLRs) are a family of transmembrane proteins that recognize pathogen-associated molecular patterns (PAMPs) to alert the host to their presence and elicit an immune response [42]. In rainbow trout, the presence of two isoforms of *tlr5*, one transmembrane and the other soluble, has been reported and like its ortholog in humans, they indicate the presence of bacterial flagellin and activate the protein complex NF-kB, which controls DNA transcription and cytokine production (Tsujita et al., 2004; 2006) [43,44]. NF-kB is involved in the cellular response to bacterial and viral antigens, playing a crucial role in the inducible expression of numerous genes involved in the immune and inflammatory responses [45]. Additionally, other pattern recognition receptors (PRRs) were also negatively regulated by RTTX such as the C-type lectin receptors (CLRs) *mrc1*, *clec4m*, and *cd209*. CLRs are a family of proteins, expressed mainly in myeloid cells, that participate in the recognition of a wide variety of pathogens and in the induction of innate and adaptive immune responses [46]. In salmonid fish, several *cd209* homologs have been identified and they also show differential expression after infection with viral and bacterial pathogens [47,48]. Nevertheless, evidence shows that some viruses can subvert CLRs to suppress or avoid antiviral immunity and promote infection [49,50]. These results suggest that the dysregulation of the inflammatory response and the innate immune response, mediated by the suppression of various PRRs genes at the beginning of the IPNV infection with RTTX, may be related to the higher mortality levels caused by this isolate in rainbow trout.

Finally, Gene Ontology analysis also indicated that several functions enriched among the differentially expressed genes in IPNV challenged trout throughout the trial were related to the regulation of metabolic processes, mainly lipid. These were found to be enriched in genes positively and negatively regulated by both RTTX and ALKA at different times post-challenge, indicating that both strains could alter the metabolism of host cells. This agrees with observations from previous transcriptomic studies with IPNV in Atlantic salmon, where gene enrichment analysis revealed massive (especially downregulation) of metabolism-related functions as a result of virus infection [28,29]. Viruses clearly depend on the energy and machinery of the host cell to be able to replicate and continue their infection process. Therefore, it is not surprising that viral infection triggers a metabolic reprogramming in host cells to generate an optimal environment that facilitates the multiplication and spread of the virus [51]. In this way, they can “hijack” cellular metabolism for their benefit by mimicking, exploiting, or interfering with the metabolic pathways of the host cell, and by doing so, they can even evade the immune response [52,53].

## 5. Conclusions

In conclusion, our results show that rainbow trout exhibit a differential transcriptomic response to infection with two IPNV isolates belonging to genogroups 1 and 5, especially during the early stage of infection. However, several functions related to enriched actin filaments were also observed between the genes regulated by both isolates, probably associated with the internalization of the virus via macropinocytosis in the cells of challenged individuals. On the other hand, the inflammatory response and the immune response were also modulated in the fish challenged with IPNV with both isolates, but with different regulation patterns throughout the challenge. These differences in the response to infection with strains other than IPNV demonstrate the great variability that exists when studying the pathogen–host interaction in viral fish diseases, and the importance of considering the phylogenetic characteristics of the viruses used in experimental challenges.

## Figures and Tables

**Table 1:** Results of the analysis by RT-qPCR to detect the presence of IPNV and estimate the viral load in the samples of trout challenged with RTTX and ALKA at days 1, 7, and 20 post-challenge. The Ct values of the test directed to the VP1 virus polymerase gene are shown. More details of the assay can be found in Tapia et al. [25].

IPNV	1 dpc	7 dpc	20 dpc
RTTX	nd	28, 5	28, 36
	nd	26, 9	28, 28
	nd	28, 5	25, 27
ALKA	nd	nd	23, 4
	nd	nd	17, 52
	nd	nd	21, 65

nd: not detected.

## Data Availability

The data presented in this study are available on request from the corresponding author.

## Supplementary Materials

The following are available online at https://www.mdpi.com/article/10.3390/v14010021/s1, Figure S1: Principal component analysis of RNA-Seq samples, Figure S2: Volcano plots of genes differentially expressed in trout challenged with the IPNV isolates RTTX (A, C, and E) and ALKA (B, D, and F) compared to uninfected controls at days 1, 7, and 20 post-challenge, Figure S3: Venn diagrams of differentially expressed genes with respect to the control in fish challenged with RTTX (WB) and ALKA (SP) at days 1, 7, and 20 post-challenge. File S1: Lists of the differentially expressed genes, Table S1: Immune response related genes differentially expressed (up- and downregulated) in fish challenged with IPNV isolates RTTX and ALKA.
