# Supplementary material for: Differential Transcriptomic Response of Rainbow Trout to Infection with Two Strains of IPNV
Source: Viruses. 2021 Dec 23;14(1):21. doi: 10.3390/v14010021 (PMC8780770; doi:10.3390/v14010021)
Supplement: Supplementary file 1 [file viruses-14-00021-s001.zip › viruses-1468424-supplementary material.pdf]

Supplementary materials

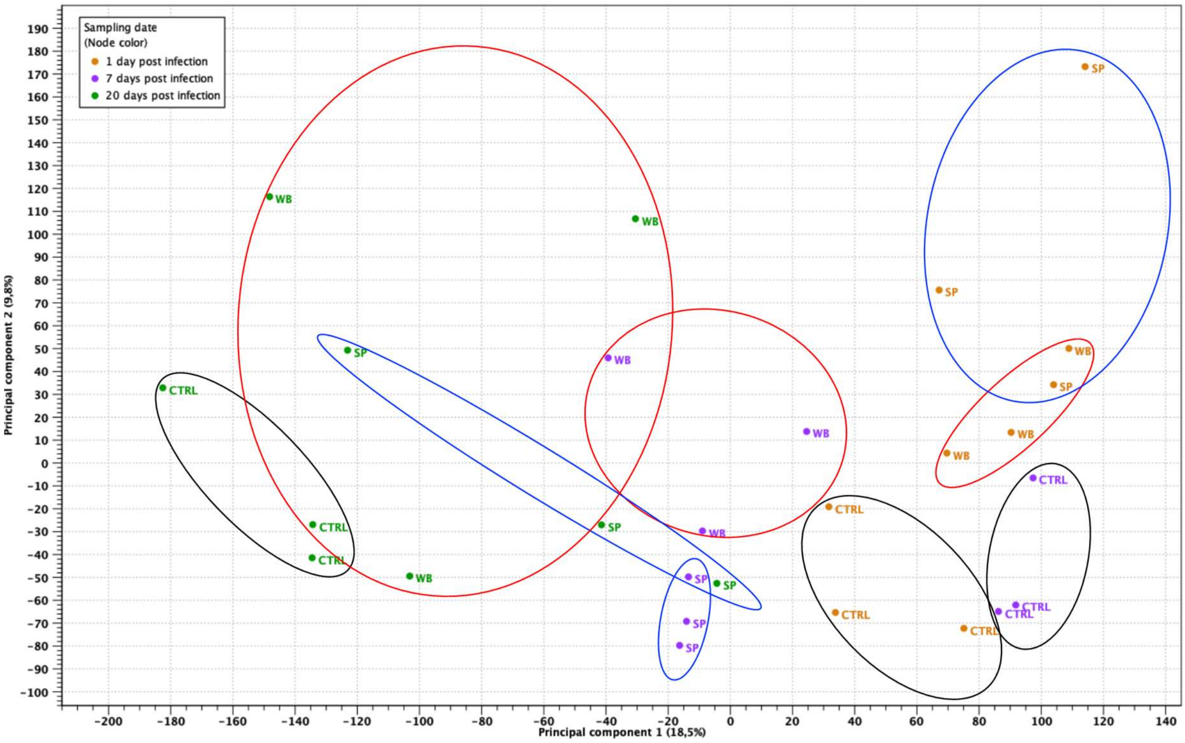

Figure S1. Principal component analysis of RNA-seq samples

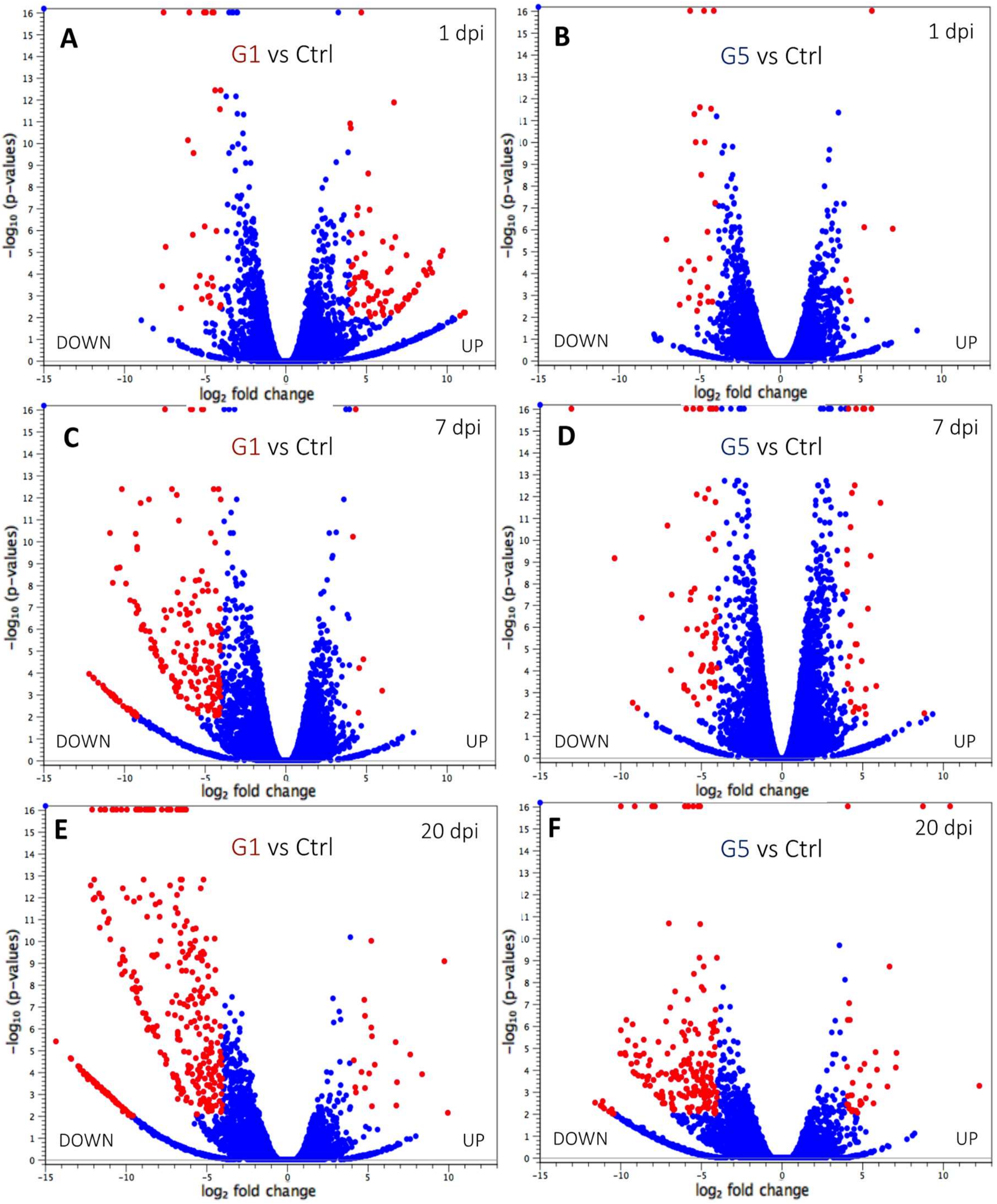

Figure S2. Volcano plots of genes differentially expressed in trout challenged with the IPNV isolates RTTX (A, C and E) and ALKA (B, D and F) compared to uninfected controls at days 1, 7 and 20 post-challenge. The red dots represent genes with significant differential expression (FDR $\leqslant$ 0.01) and a large change in absolute expression when compared to the control (LogFC $\geqslant$ |4|), while the blue dots indicate those genes that do not comply with such criteria.

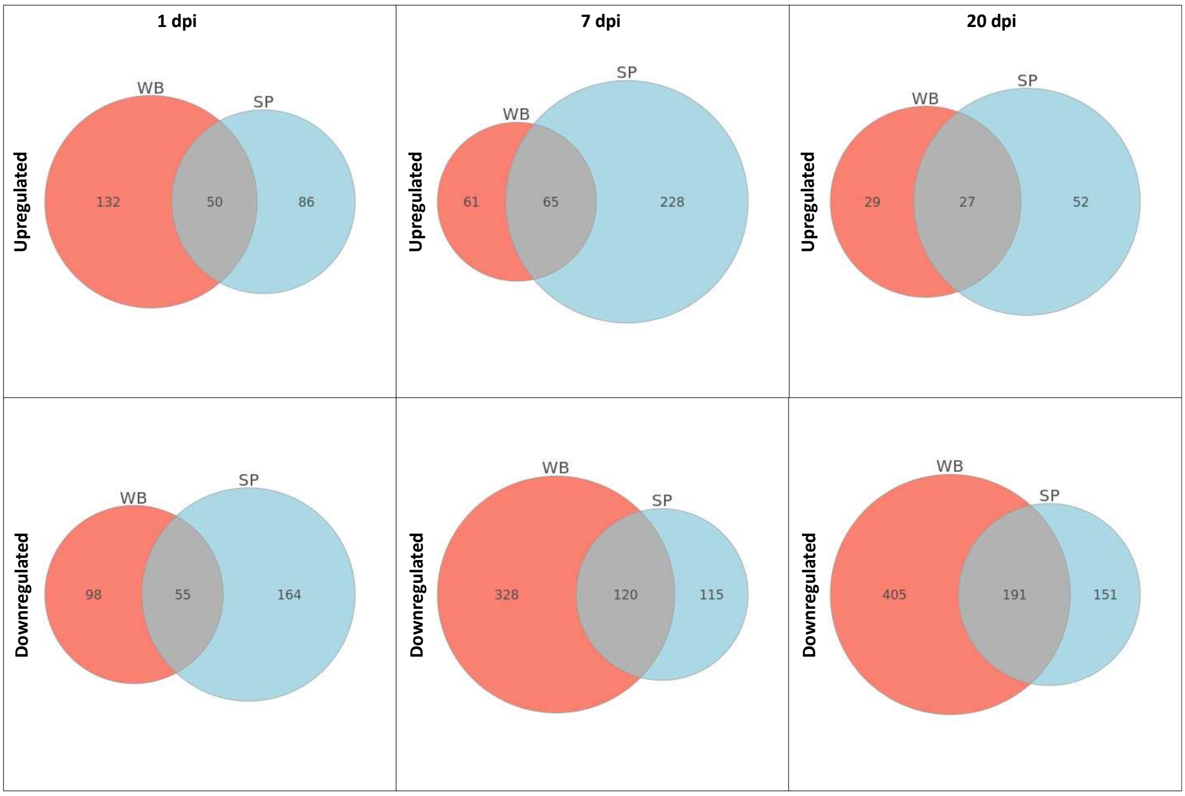

Figure S3. Venn driagrams of differentially expressed genes with respect to the control in fish challenged with RTTX (WB) and ALKA (SP) at days 1, 7 and 20 post-challenge.

Table S1. Immune response related genes differentially expressed (up and down regulated) in fish challenged with IPNV isolates RTTX and ALKA. The human homologues for each group are shown, as well as those that were common between both groups (BOTH)

Group	Regulation	1 dpc	7 dpc	20 dpc
**ALKA**	**Up**	*CD79B TRIM39 C4B_2 IGLL5 PLA2G3 AHR APOA4 MR1 CD200 PTGDS CFP TLR5 MPO CST3 ITGB7 AHSG S100A11 LEAP2 PROS1*	*TRIM21 CRP TRIM39 ZC3H12A SLC26A6 MAP3K14 EDN1 ARG2 CD209 CLEC4M AXL UMOD MX2 MRC1 PGC RGCC NFKBID FTH1 GP2 CRISP3 HEBP2 COL17A1 FOLR3 MUC5B TNFAIP6 AMPD3 TCN1 EEF2 GAA PSAP MUC5AC*	*GBP4 IGLL5 MID2 ST3GAL1 PTGDS NPPA CFHR1*
	**Down**	*CRP ZP3 ST3GAL1 ARG2 USP14 FGL2 PI4K2A HEBP2 CUL1 PSMD11 SNAP25 AMPD3 TSHB CRH S100B GYG1 PSMD12 MBP*	*TRIM35 IGLL1 TRIM39 TRIM27 APOL1 APOA4 CYP27B1 TRIM25 HLA-E CD200 PTGDS HERC5 HLA-DQB1 GCG ADGRE5*	*MEFV TRIM35 LGP2 FRK STAT1 GBP1 CRP RSAD2 IFI44L IFITM3 CCL19 TRIM25 ZC3H12A IFIT1 B2M FCER2 ZC3HAV1 PCK1 HERC5 IFIT5 MX1 PNP NLRC3 EGR1 AMPD3 MUC3A FOS SOCS3 MUC5AC*
**BOTH**	**Up**	*HHLA2 TKFC MX1 NLRC3 HEL111 MUC3A*	*TRIM35 HSP90AA1 TRIM25 SPHK2 PCK1 PNP CST3*	*HSP90AA1 FGA HEL-S-72p*
	**Down**	*HSP90AA1 ARG2 CD209 CLEC4M PCK1 MRC1 HEL-S-72p AMPD3 PSMD13*	*STAT1 HEL-S-62p RSAD2 IFI44L MFAP4 PLA2G3 APOA4 USP18 CLEC4M HERC5 IFIT5 MX1 NPPA TLR5 DNAJC3 XAF1 VCAM1 S100A11 LEAP2 MUC3A LRG1 CFHR1*	*BTN1A1 HEL-S-62p TRIM21 MFAP4 CCL19 CEACAM1 ZP4 ZC3H12A ZP3 CD209 CLEC4M IRF5 HERC5 UMOD MRC1 IFI27 MPO TIMP2 PS1TP5BP1 TCN1 EEF1A1 FOS*
**RTTX**	**Up**	*CTSV ZP4 TRIM25 ZP3 LGALS3 HK1 UMOD NPPA AKAP1 GP2 TIMP2 EEF1A1 PSAP*	*REG1B LGALS3 VCP*	*APOA4 PRF1 GP2 COL17A1 NCAM1*

**Down**	*HEL-S-62p IFITM3 MFAP4 CEACAM1 ACOD1 CYP27B1 NOS2 ARG2 C7 CLEC4E UMOD TLR5 GP2 PIK3CG HEL-S-72p ADM2 GPR84 LRG1 SOCS3*	*MEFV TRIM35 CCL20 HEL-S-62p CTSV IFI44L MFAP4 CXCL11 CCL19 TRIM39 ACOD1 ZP4 TRIM25 ZP3 CST7 IRF5 CLEC10A CLDN1 UMOD IFI27 BMP6 ALDH3B1 HEL57 TIMP2 PS1TP5BP1 MMP9 CAND1 GPR45 EEF1A1 HP*	*RIPK2 CD274 CTSL CTSV MFAP4 TRIM39 AHR ZP4 ZP3 CST7 CD209 CLEC10A GSN HPRT1 UMOD TLR5 MAP3K5 AKAP1 LRP1 NLRC3 FTH1 DLL1 ALDH3B1 HEL57 TIMP2 PS1TP5BP1 LYZ RAPGEF4 SYNGR1 MMP9 GLA NPDC1 PADI2 CTSD XAF1 GYG1 APEH TSHR PSMC3 ADGRG3*
